# Effects of health-information-based diabetes shared care program participation on preventable hospitalizations in Taiwan

**DOI:** 10.1186/s12913-019-4738-1

**Published:** 2019-11-27

**Authors:** Yia-Wun Liang, Hsiao-Feng Chang, Yu-Hsiu Lin

**Affiliations:** 1 Department of Senior Citizen Services Management, National Taichung University of Science and Technology, Taichung, Taiwan, Republic of China; 2 Department of Nursing, Chung-Kang Branch, Cheng Ching Hospital, Taichung, Taiwan, Republic of China; 3 Graduate Institute and Department of Information Management/Graduate Institute of Healthcare Information Management, Center for Innovative Research on Aging Society (CIRAS), 168, Sec. 1, University Road, Min-Hsiung, Chiayi, 62102 Taiwan, Republic of China

**Keywords:** Health information system, Diabetes shared care program, Participation, Preventable hospitalizations

## Abstract

**Background:**

Taiwan’s Diabetes Shared Care Program has been implemented since 2012, and the health information system plays a vital role in supporting most services of this program. However, little is known regarding the effectiveness of this information-based program. Therefore, this study investigated the effects of the participation of the Diabetes Shared Care Program on preventable hospitalizations.

**Methods:**

This longitudinal study examined the data of health-care claims from 2011 to 2014 obtained from the diabetes mellitus health database. Patients with diabetes aged ≥18 years were included. Preventable hospitalizations were identified on the basis of prevention quality indicators developed for administrative data by the US Agency for Healthcare Research and Quality. A multilevel logistic regression was performed to examine the effects of the participation of the Diabetes Shared Care Program on preventable hospitalizations after adjustment for other variables. Analyses were conducted in late 2018.

**Results:**

A medium level of participation (*p* = 0.05), age between 40 and 64 years(*p* < 0.0001), and absence of a catastrophic illness(*p* < 0.0001) were associated with a lower probability of having a preventable hospitalization. Male sex(*p* < 0.0001), age ≥ 65 years(*p* = 0.0203), low income level(*p* < 0.0001), living in the Southern division(*p* = 0.0106), and presence of many comorbidities(*p* < 0.0001) were associated with a higher probability of having a preventable hospitalization after adjustment for characteristics at the individual and county levels.

**Conclusions:**

The health information system records patients’ medical history, monitors quality of care, schedules patient follow-ups, and reminds case managers to provide timely health education. This health-information-based Diabetes Shared Care Program is associated with better quality care of ambulatory, so it should be promoted on a broader scale.

## Background

Obesity is a known risk factor for diabetes. In 2016, 41.1% of adults aged ≥20 years were overweight or obese in Taiwan, and the prevalence rate of diabetes in adults was 11.6% (approximately 1.4 million people) [1]. Diabetes was the fifth leading cause of death in Taiwan in 2017, resulting in a total of 9845 deaths [2]. The cost of care for patients with diabetes accounted for 3–4.24% of Taiwan National Health Insurance (TNHI) program expenditures from 1986 to 2017. The total medical expenditure for diabetes in 2017 was NT$ 29.7 billion (approximately US$ 1 billion) [2, 3].

Taiwan is a single-payer health insurance and social insurance system, as known as TNHI, organized by the government under the jurisdiction of the Ministry of Health and Welfare, Taiwan, since 1995. The TNHI covers over 99.6% Taiwanese population, and contrasts with 93% health care facilities, including hospitals, Western medicine, traditional Chinese medicine, dentist clinics, et cetera. The TNHI offers inpatient and ambulatory care, dental services, traditional medicine therapies, child delivery services, physical rehabilitation, home care, and chronic mental illness care [4].

Taiwan’s Ministry of Health and Welfare initiated the Diabetes Shared Care Program (DSCP) in 2001 to reduce the medical costs of diabetes as well as improve diabetes management and glycemic control. The Taiwan Diabetes Shared Care System is based on a chronic model with multi-disciplinary care team and provides financial incentives for providers to increase regular follow-up visit, self-education, and comprehensive diabetes-specific assessment [5]. The DSCP team members, including physicians, nurses, dietitians, are required to participate in clinical training to become certified in Taiwan Diabetes Shared Care System [5]. The physicians conduct the patients’ care plans as case managers. The rates of joining DSCP in Taiwan is variety by healthcare settings in 2018: 100% in medical centers (*n* = 19), 92.6% in regional hospitals (*n* = 75), 53.3% in district hospitals (*n* = 179), 12.5% in clinics (*n* = 780), and 70.8% in health centers (*n* = 225) [6]. The percentage of patients treated under the DSCP increases from 23.52% in 2005 to 44.60% in mid-2016 [4].

Under the DSCP, during patients’ initial visit, physicians would review patients’ conditions, when the patient met the criteria the physician could decide whether the patient enrolls in their program or not, as well as the patient could opt in or not. After patients enrolled the program, medical history is obtained, a physical examination and laboratory evaluation are conducted, a diabetes management plan is prepared, and diabetes self-management education is provided. The examination of continued care and annual follow-ups will focus more on evaluation sections and based upon the baseline assessment (the details of items are provided in Additional file 1: Table S1) [7]. Hickman defined shared care as “the joint participation of hospital consultants and general practitioners in the planned delivery of care for patients with a chronic condition, informed by an enhanced information exchange over and above routine discharge and referral notes.” [8] Shared care also emphasizes multidisciplinary teamwork in diabetes management, including treating physicians, diabetes specialists, nurses, and dietitians [9]. Following the implementation of the DSCP, the standardized mortality rate in the diabetes population decreased from 39.8 in 2001 to 26.9 in 2011 [1]. Therefore, the DSCP became a financial incentive program, which is based on pay-for-performance, in the TNHI in 2012.

The DSCP is based on a health information system. The National Health Insurance Administration, Ministry of Health and Welfare, hosts the National Health Insurance Information System Service (NHIISS), which is based on a virtual private network. For security concerns, the NHIISS uses the hypertext transfer protocol secure, and records patients’ medical history, monitors care quality, conducts patient follow-ups, and provides case management services. Hospitals may have their own health information systems for managing their patients; however, they are required to report the aforementioned information to the NHIISS. The National Health Insurance Administration monitors care quality indicators, namely the participation rate of the DSCP, HbA1c levels (< 7.0 and > 9.0% indicate favorable and poor glycemic control, respectively), and low density lipoprotein levels (< 100 and > 130 mg/dL indicate favorable and poor control, respectively). TNHI also pays extra bonuses based on the quality indicators for hospitals/clinics, or on the rate of new case and the quality indicators for physicians [7].

Moreover, if a patient is enrolled in the DSCP in hospital A, then that patient cannot enroll in the DSCP in hospital B because the NHIISS connects the hospital information system to constitute a single registry for patients in hospitals.

Ambulatory care-sensitive conditions (ACSCs) offer a valuable perspective on system performance and thus can be used to evaluate primary care physicians’ access, availability, and effectiveness [10–12]. The US Agency for Healthcare Research and Quality (AHRQ) developed prevention quality indicators, which are defined by the ICD-9-CM, to identify ACSCs [13]. Since 1993, the Institute of Medicine has recommended using ACSCs to monitor access of care [12]. Hospitalizations caused by ACSCs are considered preventable hospitalizations (PHs). To date, no study has evaluated the effect of the information-based DSCP on the incidence of PH. Therefore, the present study investigated the effect of the participation of the DSCP on the incidence of PH.

## Methods

### Study sample

**Fig. 1:**
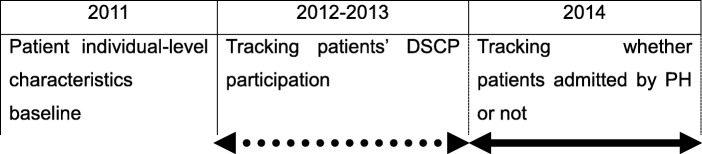
Time flow of the study

The DSCP has been a pay-for-performance program in the TNHI since 2012, and we focused on the effects observed in the first three years. Analyses were conducted in late 2018. This longitudinal study included analyses at individual and county levels. Data for the individual-level analysis were obtained from the 2011–2014 Diabetes Mellitus Health Database (DMHD), which is based on the nationwide Taiwan National Health Insurance Research Database. All data are deidentified and encrypted to protect participants’ privacy. All patients in the DMHD were diagnosed with Type I and Type II diabetes (ICD-9-CM code 250). The exclusion criteria include age ≤ 18, gestational diabetes mellitus, and missing data. In this study, we used four subsets of the database: registry for beneficiaries (DM_ENROL), ambulatory care expenditures by visits (DM_OPDTE), details of ambulatory care order (DM_OPDTO), and inpatient expenditures by admissions (DM_IPDTE). County-level data were retrieved from the 2014 Taiwan Hospital and Clinic Statistics. The time flow of this study is displayed in Fig. 1.

Firstly, merging 2011–2014 DM_ENROL, DM_OPDTE, DM_OPDTO, and DM_IPDTE, a total 404,418 people included in the merged dataset (noted: not everyone contains 4-year claim data). Secondly, in accordance with the study purpose, only the data of adults aged ≥18 years, diagnosed as type I or II diabetes, and contained 2011–2014 claims were collected, and remained 61,032 people in the analytic dataset. Finally, after disregarding missing values and outliers in the study variables and merging county-level data, a total of 60,962 patients from 22 counties were included in this study. Approval for the analysis of the database was obtained from the Institutional Review Board of Chung Kang Branch, Cheng Ching Hospital, Taiwan (IRB No. HP180005).

## Measures

### Dependent variables

We used definitions from the AHRQ for diagnosing ACSCs. ACSCs for adults included specific diagnoses of asthma, angina, congestive health failure, bacterial pneumonia, chronic obstructive pulmonary disease, dehydration, long-term and short-term diabetes complications, hypertension, lower-extremity amputation for patients with diabetes, perforated appendix, uncontrolled diabetes, and urinary tract infection [13]. Hospitalization for any of these diagnoses was considered hospitalization for an ACSC, also known as a PH.

### Key explanatory variables

#### Participation of the DSCP

The participation of the DSCP is divided into four levels: none, low, medium, and high. None indicates that the patient did not enrolled in the DSCP; low participation indicates that the patient only enrolled in the DSCP or followed up, but without annual exam (procedure code: P1407C and P1408C); medium participation indicates that the patient completed the first stage of the DSCP (three follow-ups and an annual review) (procedure code: P1409C); and high participation indicates that the patient began the second stage of the DSCP follow-up or completed the second stage annual review (procedure code: P1410C and P1411C).

### Covariates

Covariates potentially associated with PHs included characteristics at individual and county levels. Individual-level characteristics included patients’ sociodemographic and health-related variables. Sociodemographic variables included sex, income level, and region of residence. Region of residence was defined according to patients’ health insurance administration division, namely Taipei, Northern, Central, Southern, Kaoping, and Eastern divisions. Health-related variables included comorbidities and catastrophic illnesses. A Charlson comorbidity score was calculated for each patient to measure the comorbidities [14]. Catastrophic illnesses are approved by the Bureau of National Health Insurance, including 29 catastrophic illnesses, such as malignant neoplasm, systemic lupus erythematosus, etc., and other rare diseases. Patients with catastrophic illnesses certificates can apply catastrophic illness registration cards, and eligible for exemption from insurance premiums and copayments.

County-level characteristics were represented by health-care resources and health-care personnel density. Health-care resources included the number of general hospitals and clinics. The number of physicians represented health personnel density. County-level characteristics were adjusted by population size and were calculated as the number of each variable divided by total population in the area multiplied by 100,000. County-level characteristics were divided into two groups based on the mean score: low (deprived) and high (affluent).

### Statistical analysis

Descriptive data analysis was performed for individual-level characteristics. Chi-square and independent t tests were used to examine bivariate correlations between each individual-level characteristic and PHs. The data had a hierarchical structure, in which individual data (level 1) were nested within counties (level 2). A multilevel analysis was performed to control for the county effect on PH. A random intercept multilevel model was preferred over other statistical approaches because it tested whether the participation of the DSCP was associated with PHs among patients across counties. Regression coefficients and variance components at county and individual levels were estimated for PHs.

Three models were fitted. The first model in the output was an empty model; that is, a model with no predictors. The empty model was used to determine whether the overall difference between counties and individuals in terms of PHs would be significant. The second model included only individual-level variables, and the third model included both individual-level and county-level variables. A multilevel logistic regression was performed to estimate adjusted odds ratios (ORs) with 95% confidence intervals (CIs) and *p* values. Dependent variables used in multilevel logistic regression models were dichotomous; patients with PH were coded as 1, and those without PH were coded as 0. The equation for the multilevel logistic model is as follows:

$$ \mathrm{logit}\left({\pi}_{ij}\right)=\alpha +{u}_j+{\beta}^{\tau }{X}_{ij} $$

where ${u}_j\sim \mathrm{N}\left(0,{\sigma}_u^2\right)$, *u*_*j*_ is the random effect and *j* represents county-level characteristics. α and β are fixed effects, α represents the intercept, and *i* represents individual-level characteristics.

Intraclass correlation coefficients (ICCs) were calculated to determine the contribution of variance at the county level to the total variance. For multilevel linear models, the ICC was calculated using the following formula:

$$ \mathrm{ICC}=\frac{\sigma_n^2}{\sigma_i^2+{\sigma}_n^2} $$

where ${\sigma}_n^2$ = county-level variance and ${\sigma}_i^2$ = individual-level variance. Because the variance of a logistic distribution with a scale factor of 1 is π^2^/3 (approximately 3.29) in a hierarchical logistic regression model, this formula can be reformulated as follows [15]:

$$ \mathrm{ICC}=\frac{\sigma_n^2}{\sigma_n^2+\left(\frac{\uppi^2}{3}\right)} $$

All statistical analyses were performed using SAS version 9.4 (SAS Institute, Inc., Cary, NC, USA). Statistical significance was determined for differences with a two-sided *p* value of < 0.05.

## Results

### Description of the study population

About one-sixth (15.79%) of 9624 adults had PHs. The average age of patients was 64.20 (*SD* = 14.25) years. Only 7.48% of patients completed the first stage DSCP (both medium and high participation), and 71.76% of patients were not enrolled in the DSCP in 2012–2013. Table 1 presents the sociodemographic characteristics and health-related factors of patients.

**Table 1:** Descriptive and bivariate correlation analyses, individual-level (*n* = 60,962)

	Preventable hospitalization n/Mean ± Std.		n(%)/Mean ± Std.	Chi-square/t-value(*p*)
	No (51,338, 84.21%)	Yes (9624, 15.79%)		
DSCP participation				
None	41,925	7916	49,841(71.76)	2.48(0.480)
Low	5543	1016	6559(10.76)	
Medium	3810	679	4489(7.36)	
High	60	13	73(0.12)	
Sociodemographic characteristics				
Gender				
Female	28,580	4987	33,567(55.06)	**48.60(< 0.001)**^**a**^
Male	22,758	4637	27,395(44.94)	
Age	63.66 ± 13.88	67.05 ± 15.78	64.20 ± 14.25	**−21.47(< 0.001)**^**b**^
19–40	2842	562	3404(5.58)	**357.95(< 0.001)**^**a**^
41–64	24,456	3590	28,046(46.01)	
65+	24,040	5472	29,512(48.41)	
Income level				
Non-low income level	50,178	9349	59,527(97.65)	**12.61(< 0.001)**^**a**^
Low income level	1160	275	1435(2.35)	
Living region				
Taipei division	16,590	2772	19,362(31.76)	**69.46(< 0.001)**^**a**^
Northern division	6584	1321	7905(12.97)	
Central division	9158	1862	11,020(18.08)	
Southern division	8279	1742	10,021(16.44)	
Kaoping division	8918	1589	10,507(17.24)	
Eastern division	1809	338	2147(3.52)	
Health related factors				
Comorbidity	0.43 ± 0.87	0.50 ± 0.93	0.44 ± 0.88	**−6.79(< 0.001)**^**b**^
Catastrophic illness				
No	47,417	9057	56,474(92.64)	**36.23(< 0.001)**^**a**^
Yes	3921	567	4488(7.36)	

Note: ^a^ Chi-square test; ^b^ independent t-test. Boldface indicates statistical significance (*p* < 0.05)

Table 1 lists the estimates of bivariate analyses performed to identify group differences across variables. The incidence of PH varied significantly with sociodemographic characteristics and health-related factors; for example, it differed significantly between sexes, with men having a higher incidence of PH.

### Multilevel assessment of factors associated with preventable hospitalization

The following are the results of multilevel models using the incidence of PH as the dependent variable. In the first model, approximately 0.575% of variance in the incidence rate of PHs was accounted for at the county level; thus, the remaining 99.425% of variance was accounted for at the individual level or by other unknown factors. These results also indicated that there is a statistically significant amount of variance in the log odds of having a PH among the counties included in the sample (τ_00_ = 0.0190; z = 2.66, *p* = 0.0039). In the unconditional model (model 1), the probability of having a PH at a typical county was 0.20; however, the probability of having a PH varied considerably across counties (Table 2).

**Table 2:** Multilevel logistic regression models for the probability of having a preventable hospitalization, 2011–2014

	Model 1 Empty model	Model 2 individual-level	Model 3 both individual- and county-level
	Estimate(SE)	Estimate(SE)	Estimate(SE)
Intercept	−1.62(0.03)^***^	−1.69(0.06)^***^	−1.62(< 0.001)^***^
Individual-level			
DSCP participation			
None(ref)			
Low		−0.01(0.04)	−0.01(0.04)
Medium		−0.09(0.04)^*^	−0.09(0.04)^*^
Full		0.29(0.81)	0.35(0.80)
Sociodemographic characteristics			
Gender			
Female(ref)			
Male		0.13(0.02)^***^	0.13(0.02)^***^
Age			
19–40(ref)			
41–64		−0.30(0.05)^***^	−0.30(0.05)^***^
65+		0.12(0.05)^*^	0.11(0.05)^*^
Income level			
Non-low income level(ref)			
Low income level		0.34(0.07)^***^	0.34(0.07)^***^
Living region			
Taipei division(ref)			
Northern division		0.06(0.05)	0.07(0.05)
Central division		0.08(0.05)	0.09(0.05)
Southern division		0.12(0.05)^*^	0.13(0.05)^*^
Kaoping division		0.06(0.06)	0.07(0.06)
Eastern division		−0.03(0.09)	−0.03(0.09)
Health related factors			
*Comorbidity*		0.08(0.01)^***^	0.08(0.01)^***^
*Catastrophic illness*			
No(ref)			
Yes		−0.27(0.05)^***^	−0.27(0.05)^***^
County-level: Area health resources			
Hospitals to population ratio			
Low(ref)			
High			−0.02(0.06)
Physicians to population ratio			
Low(ref)			
High			−0.07(0.07)
Clinics to population ratio			
Low(ref)			
High			−0.09(0.07)
Variance (s.e.)	0.0190 (0.0071)	0.0145 (0.0060)	0.00987 (0.0044)
z value for covariance parameter estimates	2.66^**^	2.44^**^	2.26^*^
ICC%	0.575%	0.439%	0.299%
-2 Log Likelihood	53,040.48	52,563.17	52,557.70
AIC	53,044.48	52,595.17	52,595.70
AICc	53,044.49	52,595.18	52,595.71
BIC	53,046.67	52,612.62	52,616.43
Pearson Chi-Square	60,878.27	60,908.46	60,913.13
Pearson Chi-Square/DF	1.00	1.00	1.00

Note: Boldface indicates statistical significance (*p* < 0.05). *p < 0.05, **p < 0.01, ***p < 0.001. Values based on SAS PROC GLIMMIX. Entries show parameter estimates with standard errors in parentheses; Estimation Method: Laplace. Model 3 has better fit than Model 2. Ref: reference group. Observation number: 40,093

The estimated variance was 0.439% in model 2 and 0.299% in model 3. The ICC of model 3 indicated that 0.299% of variance could be attributed to the county level. The ICC of model 3 reduced the percentage of variance associated with nesting within counties by 32% in model 2 (Table 2).

In model 3, male sex (b = 0.13, *p* < 0.0001), age ≥ 65 years (b = 0.11, *p* = 0.0203), low income level (b = 0.34, *p* < 0.0001), living in the Southern division (b = 0.13, *p* = 0.0106), and presence of many comorbidities (b = 0.08, *p* < 0.0001) were associated with a higher probability of having a PH. By contrast, age between 41 and 64 years (b = − 0.30, *p* < 0.0001), presence of a catastrophic illness (b = − 0.27, *p* < 0.0001), and medium DSCP participation (b = − 0.09, *p* = 0.050) were associated with a lower probability of having a PH (Table 2).

After considering factors at both individual and county levels, patients with medium DSCP participation had an adjusted OR of 0.918 (95% CI = -0.1720, 0.000013) for having a PH compared with patients who were not enrolled in the DSCP (Table 2).

## Discussion

We performed a multilevel logistic regression by using a hierarchical model to determine the probability of having a PH. Cross-level interactions enabled the analysis of effects among different population subgroups. In general, after adding county-level factors, the coefficients of individual characteristics changed slightly. However, the obtained ICCs suggest that county-level factors may not significantly contribute to the probability of having a PH. Rather, this probability may be influenced by region of residence as an individual-level factor; this factor reduced the effect of county-level factors. In general, the Taipei division contains affluent health resources. Patients living in the Taipei division had easier access to health care and higher quality of care.

Lin, Chen, Wu, and Chen performed a data envelopment analysis to measure the effectiveness of five dimensions of equality, namely equality of access, needs, health, choice sets and expenditures, and constructs of equity [16]. On the basis of measure of effectiveness, their investigation could assess the equity condition, including the concepts of horizontal equity, healthcare to those in primary health need, and vertical equity, addressing those with the greatest need [17], and change after introducing TNHI. In their study, Taipei region had the highest effectiveness among different counties. Therefore, although we applied three county-level health resource variables, the county-level effect was still weak.

The results of this study indicated an association between DSCP participation and incidence of PHs. Diabetes may cause the development of an ACSC because its management depends heavily on outpatient services and because hospital admissions for hyperglycemia or hypoglycemia are generally preventable in patients receiving satisfactory ambulatory care [18]. Lee, Cheng, Chen, and Lai indicated that the DSCP was associated with a significant increase in regular follow-up visits and evidence-based services and significantly reduced hospitalization costs [5]. Another study reported that after a chronic disease self-management program intervention, patients had fewer occurrences and shorter durations of hospitalizations because it was possible to educate patients successfully in the same intervention at the same time [19].

An adequate care or management program could improve patients’ health status and reduce adverse health outcomes, such as cardiovascular events, stroke, all-cause mortality, and cancer-specific and diabetes-related mortality [20, 21]. Yu et al. reported that the rate of having an HbA1c level of < 7% increased from 20.9% in 2002 to 34.5% in 2011 [22]. This finding indicated that diabetes control improved after the implementation of the DSCP. In addition, the DSCP could reduce medical expenditures and improve health outcomes [23, 24]. Patients who received better clinical care and continuous care had lower rates of hospitalization after being enrolled in the DSCP. Furthermore, although the cost of outpatient visits increased by US$ 110, the cost of admission decreased by US$ 130 [5]. The results of the present study support the finding of a previous study that reported that DSCP participation in the first stage reduced adverse health outcomes and increased the quality of care [23, 24].

The American Diabetes Association publishes the “Standards of Medical Care in Diabetes” annually and provides clinical practice recommendations and intents regarding diabetes care, general treatment goals and guidelines, and tools for evaluating the quality of care [25]. According to the standards, health education plays a critical role in diabetes care, and diabetes patients should receive diabetes self-management education. The functions of DSCP in Taiwan are similar to Diabetes self-management education and support (DSMES): both are covered by health insurance plans and reimbursed when received in person, and proven to be cost-effective program by improving patient health and reducing risks of complications, hospitalization fees, total health care expenses, and mortality rate [5, 26–28]. Following reimbursement for the DSMES or DSCP, high accessibility and utilization of these programs would exert positive effects on participating patients’ clinical outcomes, quality of care, health-care utilization, and health-care costs [25].

This study shows that a medium DSCP participation was associated with a lower probability of having a PH may possibly indicate having an ongoing doctor–patient relationship is a factor associated with decreased PHs. However, additional research is needed to further assess the association. In addition, physicians will pay more attention to those diabetes patients with catastrophic illness, therefore, these patients have lower probability of having PHs.

### Limitation

This analysis was limited by several factors. First, extrapolation bias was a limitation; although patients in the DMHD were diagnosed with diabetes, we could not include all patients with diabetes from this database. In this study, we applied a stricter criterion that diabetes patients must having at least one ambulatory or inpatient visit for diabetes in 2011, which means those who were not regular outpatient visit diabetes patients or new cases after 2011 were not considered as study subjects in this study. Therefore, the probability of having a PH determined in this study cannot be generalized to other patients who were not included from the DMHD. We were unable to ascertain the extent to which this bias might prevail in other patients with diabetes. Secondly, the selection bias may occur in whether diabetes patients enrolled in the program, because patients could opt in or out it. Moreover, the low enrollment rate in this study may also result in the selection bias. Finally, the DMHD does not provide information regarding other sociodemographic and health-related factors, such as education, physical examinations, laboratory evaluations, and self-management contents. These factors may also affect the probability of having a PH.

## Conclusions

Although we adjusted for individual-level factors, contextual factors continued to exert an important effect on the incidence of PHs; however, the effects of contextual factors were generally weaker than those of individual-level factors. Individual-level characteristics exerted a stronger effect than did county-level characteristics. The results of this study provide potential implications for the provision of health and social services and more generally for policies affecting community cohesiveness. Our findings provide a basis for developing targeted intervention programs for diabetes patients, allocating resources to deprived areas, and evaluating the effects of future interventions.

Emerging evidence has demonstrated the benefit of Internet-based DSMES services for diabetes prevention and management [29–32]. Ralston et al. reported that care management delivered through secure patient web communications improved glycemic control in patients with type 2 diabetes [33]. Greenwood et al. indicated that technology-enabled diabetes self-management solutions improved HbA1c most effectively [32]. Consequently, when the DSCP is supported by an efficient health information system, it can provide better case management services to patients. The enrollment rate in the DSCP was still low in 2012, 11 years after its implementation (only 15.1% in our study). Thus, we suggest that hospitals should develop a strong health information system for DSCP case management and connect to the NHISS.

## Supplementary information

**Additional file 1:** **Table S1.** Comparisons of Components of DSCP

## Data Availability

Not applicable. The data that support the findings of this study are available from the Health and Welfare Data Science Center, Ministry of Health and Welfare, Taiwan, but restrictions apply to the availability of these data, which were used under license for the current study, and so are not publicly available. Data are however available from the author upon reasonable request and with permission of the Health and Welfare Data Science Center, Ministry of Health and Welfare, Taiwan.

## Abbreviations

ACSCs: Ambulatory care-sensitive conditions
AHRQ: US Agency for Healthcare Research and Quality
CIs: Confidence intervals
DMHD: Diabetes Mellitus Health Database
DSCP: Diabetes Shared Care Program
ICCs: Intraclass correlation coefficients
NHIISS: National Health Insurance Information System Service
ORs: Odds ratios
PHs: Preventable hospitalizations
TNHI: Taiwan National Health Insurance
