# Supplementary material for: Effects of health-information-based diabetes shared care program participation on preventable hospitalizations in Taiwan
Source: BMC Health Serv Res. 2019 Nov 27;19:890. doi: 10.1186/s12913-019-4738-1 (PMC6880543; doi:10.1186/s12913-019-4738-1)
Supplement: Supplementary file 1 — Additional file 1: Table S1. Comparisons of Components of DSCP [file 12913_2019_4738_MOESM1_ESM.docx]

**Effects of Health-Information-Based Diabetes Shared Care Program Participation on Preventable Hospitalizations in Taiwan**

Table S1: Comparisons of Components of DSCP

| Items | Initial visit | Continuing care visit | Annual exam |
| --- | --- | --- | --- |
| **Medical history** |  |  |  |
| 1. Symptoms | ✓ |  |  |
| 1. Laboratory results related to diagnosis | ✓ |  |  |
| 1. Previous and present treatment plans | ✓ |  |  |
| 1. Current treatment program | ✓ |  |  |
| 1. Exercise history | ✓ |  |  |
| 1. Acute complications | ✓ |  |  |
| 1. History of infections | ✓ |  |  |
| 1. Chronic diabetic complications | ✓ |  |  |
| 1. Medication history | ✓ |  |  |
| 1. Family history | ✓ |  |  |
| 1. CHD risk factors | ✓ |  |  |
| 1. Psychosocial/economic factors | ✓ | ✓ | ✓ |
| 1. Tobacco and alcohol use | ✓ | ✓ | ✓ |
| 1. Frequency/severity of hypo-/hyperglycemia |  | ✓ | ✓ |
| 1. SMBG results |  | ✓ | ✓ |
| 1. Patient regimen adjustments |  | ✓ | ✓ |
| 1. Adherence problems |  | ✓ | ✓ |
| 1. Lifestyle changes |  | ✓ | ✓ |
| 1. Symptoms of complications |  | ✓ | ✓ |
| 1. Other medical illness |  | ✓ | ✓ |
| **Physical examination** |  |  |  |
| 1. Height and weight | ✓ | ✓ | ✓ |
| 1. Blood pressure | ✓ | ✓ | ✓ |
| 1. Ophthalmoscopic examination | ✓ |  | ✓(dilated eye examination annually) |
| 1. Thyroid palpation | ✓ |  |  |
| 1. Cardiac examination | ✓ |  |  |
| 1. Evaluation of pluses | ✓ |  |  |
| 1. Foot examination | ✓ | ✓(if indicated) | ✓(if indicated) |
| 1. Skin examination | ✓ |  |  |
| 1. Neurological examination | ✓ |  |  |
| 1. Oral examination | ✓ |  |  |
| 1. Sexual maturation (if peripubertal) | ✓ |  |  |
| 1. Previous abnormalities on the physical exam |  | ✓ | ✓ |
| **Laboratory evaluation** |  |  |  |
| 1. Fasting plasma glucose or capillary blood sugar | ✓ | ✓ | ✓ |
| 1. HbA1C | ✓ | ✓ | ✓ |
| 1. Fasting lipid profile (cholesterol, total, triglyceride, HDL cholesterol, LDL cholesterol) | ✓ |  | ✓ |
| 1. Serum creatinine | ✓ |  | ✓ |
| 1. SGPT (or ALT) | ✓ |  | ✓ |
| 1. Urine biochemistry examination | ✓ |  | ✓ |
| 1. ACR/Microalbumin (Nephelometry) | ✓ |  | ✓ |
| 1. Urine culture (if indicated) | ✓ |  |  |
| 1. TSH (type 1 patients) | ✓ |  |  |
| 1. Electrocardiogram (adults) | ✓ |  | ✓ |
| **Management Plan** |  |  |  |
| 1. Short- and long-term goals | ✓ | ✓ | ✓ |
| 1. Medications | ✓ | ✓ | ✓ |
| 1. Medical nutrition therapy | ✓ | ✓ | ✓ |
| 1. Lifestyle changes | ✓ |  |  |
| 1. Self-management education | ✓ | ✓(skills) | ✓(skills) |
| 1. Monitoring instructions | ✓ |  |  |
| 1. Annual referral to eye specialist (if indicated) | ✓ |  |  |
| 1. Agreement on continuing support/follow-up | ✓ |  |  |
| 1. Influenza vaccine (if indicated) | ✓ | ✓ | ✓ |
| 1. Glycaemia |  | ✓ | ✓ |
| 1. Frequency/severity of hypoglycemia |  | ✓ | ✓ |
| 1. SMBG results |  | ✓ | ✓ |
| 1. Complications |  | ✓ | ✓ |
| 1. Control of dyslipidemia |  | ✓ | ✓ |
| 1. Blood pressure weight |  | ✓ | ✓ |
| 1. Exercise regimen |  | ✓ | ✓ |
| 1. Adherence to self-management |  | ✓ | ✓ |
| 1. Follow-up of referrals |  | ✓ | ✓ |
| 1. Psychosocial adjustment |  | ✓ | ✓ |
| 1. Knowledge of diabetes |  | ✓ | ✓ |
| 1. Smoking cessation (if indicated) |  | ✓ | ✓ |
| **Diabetes self-management education** | ✓ | ✓ | ✓ |

Ref: 4.

Note: The procedure codes for different levels of DSCP: P1407C: first stage initial visit; P1408C: first stage continuing care visit; P1409C: first stage annual exam; P1410C: second stage continuing care visit; and P1411C: second stage annual exam. When patients received at least one time initial visit, five times continuing care visit, and two times annual exam, and had better self- management, the physicians could decide whether referral to the second stage program.
